# Cellular stress induces non-canonical activation of the receptor tyrosine kinase EphA2 through the p38-MK2-RSK signaling pathway

**DOI:** 10.1016/j.jbc.2023.104699

**Published:** 2023-04-12

**Authors:** Yue Zhou, Ryota Oki, Akihiro Tanaka, Leixin Song, Atsushi Takashima, Naru Hamada, Satoru Yokoyama, Seiji Yano, Hiroaki Sakurai

**Affiliations:** 1 Department of Cancer Cell Biology, Faculty of Pharmaceutical Sciences, University of Toyama; Toyama, Japan; 2 Department of Respiratory Medicine, Faculty of Medicine, Institute of Medical, Pharmaceutical, and Health Sciences, Kanazawa University, Kanazawa, Japan; 3 Division of Medical Oncology, Cancer Research Institute, Kanazawa University, Kanazawa, Japan; 4 WPI Nano Life Science Institute, Kanazawa University, Kanazawa, Japan

**Keywords:** EphA2, RSK, p38, MK2, cellular stress, migration

## Abstract

The receptor tyrosine kinase ephrin type-A receptor 2 (EphA2) is overexpressed in malignant tumors. We previously reported that non-canonical EphA2 phosphorylation at Ser-897 was catalyzed by p90 ribosomal S6 kinase (RSK) *via* the MEK-ERK pathway in ligand- and tyrosine kinase-independent manners. Non-canonical EphA2 activation plays a key role in tumor progression; however, its activation mechanism remains unclear. In the present study, we focused on cellular stress signaling as a novel inducer of non-canonical EphA2 activation. p38, instead of ERK in the case of epidermal growth factor signaling, activated RSK-EphA2 under cellular stress conditions, including anisomycin, cisplatin, and high osmotic stress. Notably, p38 activated the RSK-EphA2 axis *via* downstream MAPK-activated protein kinase 2 (MK2). Furthermore, MK2 directly phosphorylated both RSK1 Ser-380 and RSK2 Ser-386, critical residues for the activation of their N-terminal kinases, which is consistent with the result showing that the C-terminal kinase domain of RSK1 was dispensable for MK2-mediated EphA2 phosphorylation. Moreover, the p38-MK2-RSK-EphA2 axis promoted glioblastoma cell migration induced by temozolomide, a chemotherapeutic agent for the treatment of glioblastoma patients. Collectively, the present results reveal a novel molecular mechanism for non-canonical EphA2 activation under stress conditions in the tumor microenvironment.

Ephrin type-A receptor 2 (EphA2), a member of the receptor tyrosine kinase family, plays a critical role in modulating intercellular junctions by associating with its membrane-bound Ephrin ligands in normal epithelial cells (1, 2, 3, 4, 5). Ligand binding promotes the tyrosine kinase activity of EphA2, which induces the autophosphorylation of specific intracellular tyrosine residues. Canonical EphA2 activation suppresses ERK and AKT activities, thereby attenuating cell motility and the formation of appropriate tight junctions. EphA2 is often overexpressed in various cancer cells, including breast cancer, lung cancer, and glioblastoma multiforme (3, 4, 5). In contrast, the expression of its ligands in tumor tissues is suppressed, which prompted us to characterize the tyrosine kinase-independent function of EphA2 in the tumor microenvironment (3, 6). We previously reported that p90 ribosomal S6 kinase (RSK) directly catalyzed EphA2 phosphorylation at Ser-897 independent of its tyrosine kinase activity and ligand binding (7). Although non-canonically Ser-phosphorylated EphA2 (pS-EphA2) has been implicated in malignant progression, including cancer cell motility, epithelial-to-mesenchymal transition (EMT), the maintenance of stemness properties, and drug resistance, its activation mechanisms have not yet been elucidated in detail (3, 7, 8, 9, 10).

RSK consists of four isoforms (RSK1-4) and has a unique structure harboring two distinct kinase domains: the carboxyl-terminal kinase (CTK) domain (CTKD) and amino-terminal kinase (NTK) domain (NTKD) (11, 12, 13). The activation mechanism is conserved in all isoforms, in which ERK is the responsible upstream kinase. In the case of RSK1, ERK binds to the carboxyl-terminal region and directly activates CTK *via* phosphorylation at Thr-573. CTK-mediated phosphorylation at Ser-380 in the linker region between CTKD and NTKD provides a docking site for 3-phosphoinositide-dependent kinase 1 (PDK1), which phosphorylates NTKD at Ser-221 and causes NTK-mediated downstream signaling. The phosphorylation of Thr-732 by NTK, in turn, dissociates ERK, which eventually inactivates NTK *via* the dephosphorylation of Ser-380 and Ser-221 (Fig. S1). A large proportion of driver oncogene products are known to constitutively activate the ERK-RSK pathway; therefore, constitutive non-canonical EphA2 activation often occurs in various types of human cancer cells (3, 7).

We previously reported that EphA2 at Ser-897 was phosphorylated *via* the canonical ERK-RSK pathway upon stimulation with epidermal growth factor (EGF) (7). Stress-responsive p38 mitogen-activated protein kinase has been shown to promote cell survival, motility, and resistance to chemotherapeutic agents (14, 15); however, its role in non-canonical EphA2 activation remains unclear. Therefore, we herein attempted to verify the hypothesis of crosstalk between pS-EphA2 and p38.

## Results

### The phosphorylation of EphA2 at Ser-897 is induced by cellular stress *via* p38

**Figure 1:**
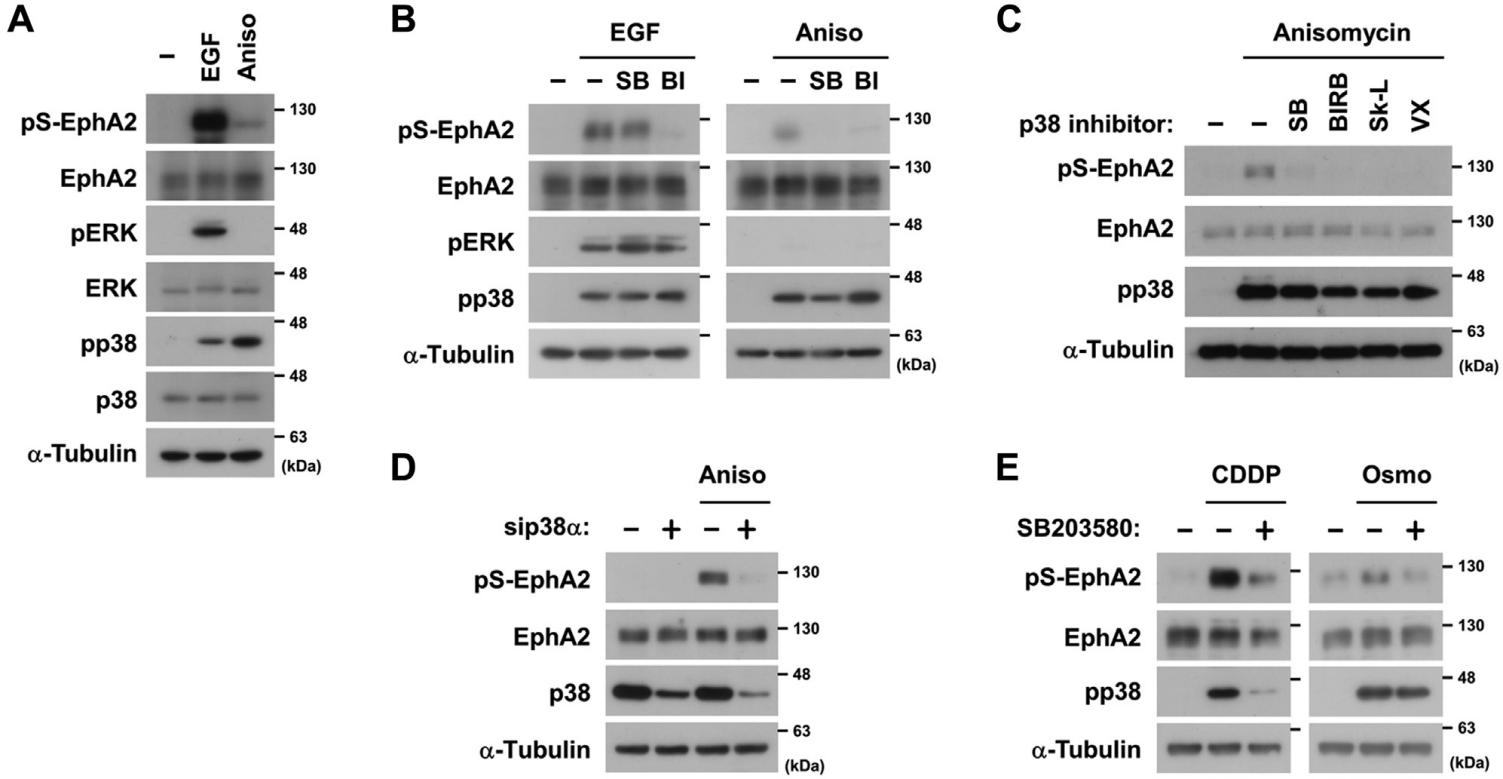
**The phosphorylation of EphA2 at Ser-897 is induced by cellular stress *via* p38.***A*, HeLa cells were stimulated with 10 ng/ml EGF for 10 min or 50 μM anisomycin (Aniso) for 20 min. *B* and *C*, HeLa cells were treated with 10 μM SB203580 (SB), BI-D1870 (BI), BIRB796 (BIRB), Skepinone-L (Sk-L), or VX-702 (VX) for 30 min and then stimulated with 10 ng/ml EGF for 10 min or 50 μM anisomycin for 20 min. *D*, HeLa cells were transfected with siRNAs against p38α or the negative control. At 72 h post-transfection, cells were stimulated with 50 μM anisomycin for 20 min. *E*, HeLa cells were treated with 10 μM SB203580 and then stimulated with 100 μM cisplatin (CDDP) for 3 h or 0.3 M NaCl (Osmo) for 10 min. whole-cell lysates were immunoblotted with primary antibodies against phospho-EphA2 at Ser-897 (pS-EphA2), EphA2, phospho-ERK (pERK), ERK, phospho-p38 (pp38), p38, and α-Tubulin. EphA2, ephrin type-A receptor 2.

To investigate whether p38 is involved in EphA2 phosphorylation at Ser-897, HeLa cells were stimulated with EGF or anisomycin. As previously reported (7), EGF induced the phosphorylation of ERK and EphA2 (Fig. 1*A*). Anisomycin, a protein synthesis inhibitor that potentially activates p38, also promoted EphA2 phosphorylation despite no significant activation of ERK. The RSK inhibitor BI-D1870 blocked both EGF- and anisomycin-induced EphA2 phosphorylation (Fig. 1*B*), whereas the p38 inhibitor SB203580 selectively inhibited the response to anisomycin. Other p38 inhibitors with different inhibition profiles exerted similar suppressive effects (Fig. 1*C*). In addition, the knockdown of p38α, the main isoform in HeLa cells, attenuated EphA2 phosphorylation (Fig. 1*D*). The platinum-based drug cisplatin (CDDP) or high osmotic stress with 300 mM sodium chloride also induced p38-mediated EphA2 phosphorylation (Fig. 1*E*). Collectively, our results demonstrated the critical involvement of p38 in the non-canonical phosphorylation of EphA2 under cellular stress conditions.

### p38 induces RSK phosphorylation to regulate EphA2 phosphorylation

**Figure 2:**
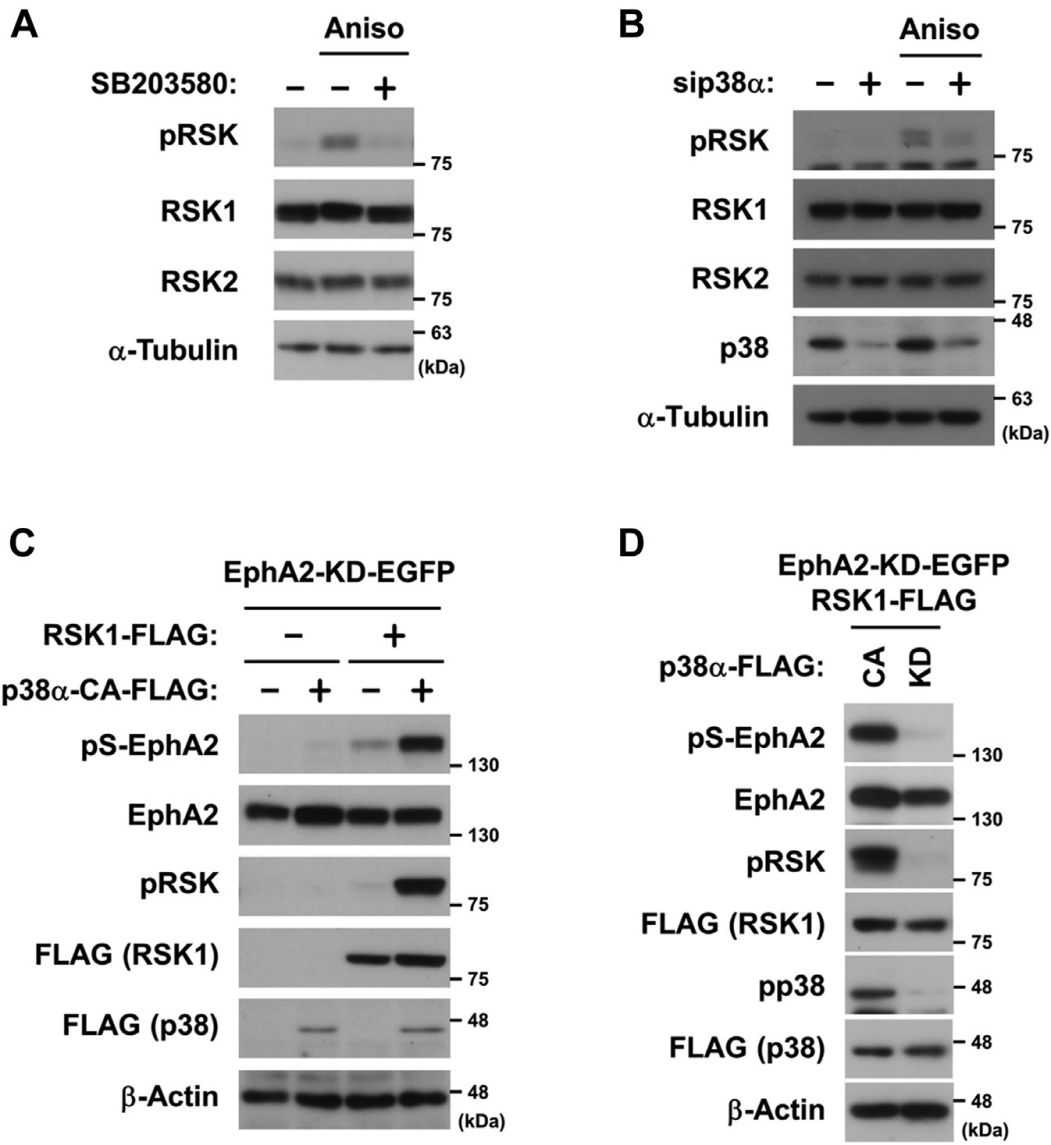
**p38 induces RSK phosphorylation to regulate the phosphorylation of EphA2 at Ser-897.***A* and *B*, HeLa cells were treated with 10 μM SB203580 for 30 min (*A*) or transfected with siRNAs against p38α or the negative control and cultured for 72 h (*B*). Cells were stimulated with 50 μM anisomycin for 20 min. Whole-cell lysates were immunoblotted with primary antibodies against pRSK, RSK1, RSK2, p38, and α-Tubulin. *C* and *D*, HEK293 cells were transfected with expression vectors for EGFP-tagged kinase-dead EphA2 (EphA2-KD-EGFP), FLAG-tagged RSK1, FLAG-tagged p38α (p38α-FLAG; constitutively activated (CA) or kinase-dead (KD) mutant), and/or an empty vector. At 24 h post-transfection, whole-cell lysates were immunoblotted with primary antibodies against pS-EphA2, EphA2, pRSK, pp38, p38, FLAG, and β-Actin. EphA2, ephrin type-A receptor 2; RSK, p90 ribosomal S6 kinase.

The anisomycin-induced phosphorylation of EphA2 was regulated by RSK (Fig. 1*B*); therefore, we investigated whether p38 activated RSK *via* phosphorylation at Ser-380. Anisomycin-induced RSK activation was clearly blocked by SB203580 or the knockdown of p38α (Fig. 2, *A* and *B*). In HEK293 cells transfected with RSK1 and kinase-dead EphA2 (EphA2-KD), only the weak phosphorylation of EphA2 was detected, whereas the phosphorylation of both RSK1 and EphA2 was strongly enhanced by the co-expression with constitutively active p38α (p38α-CA (16)) (Fig. 2*C*). In contrast, kinase-dead p38α (p38α-KD) did not induce the phosphorylation of RSK1 or EphA2, suggesting that p38 activity was essential for RSK1-mediated EphA2 phosphorylation (Fig. 2*D*). The phosphorylation of EphA2-KD indicated that its tyrosine kinase activity was not required for p38-mediated Ser-897 phosphorylation. These results showed that p38α mediated the stress-induced non-canonical RSK-EphA2 signaling pathway.

### MAPK-activated protein kinase 2 directly catalyzes RSK phosphorylation

**Figure 3:**
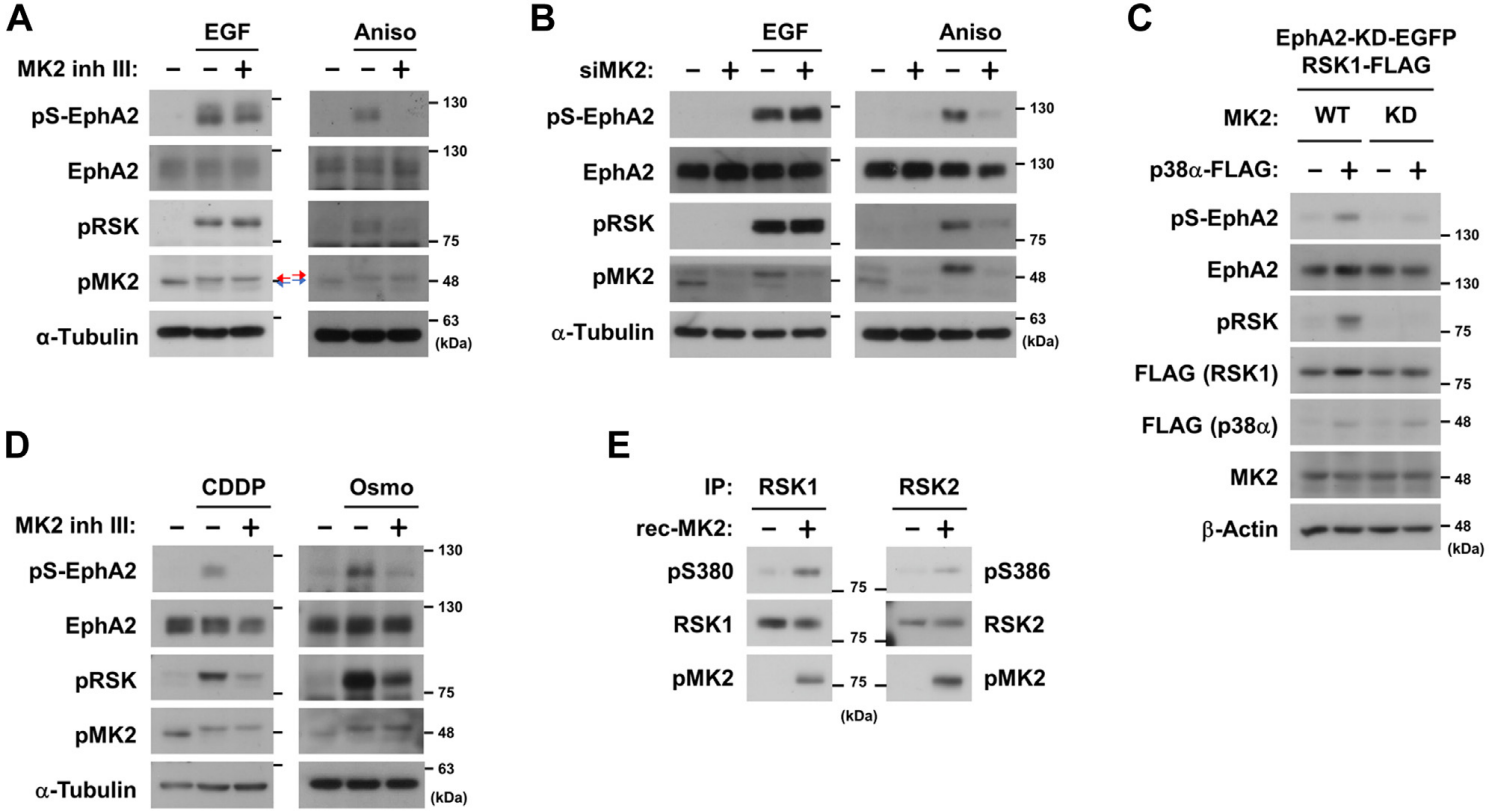
**MK2 catalyzes RSK1 phosphorylation at Ser-380.***A*, HeLa cells were treated with 10 μM MK2 inhibitor III (MK2 inh III) for 30 min and then stimulated with 10 ng/ml EGF for 10 min or 50 μM anisomycin for 20 min. *B*, HeLa cells were transfected with siRNA against MK2 or the negative control. At 48 h post-transfection, cells were stimulated with 10 ng/ml EGF for 10 min or 50 μM anisomycin for 20 min. *A* and *B*, Whole-cell lysates were immunoblotted with primary antibodies against pS-EphA2, EphA2, pRSK, phospho-MK2 (pMK2), and α-Tubulin. *Red arrow*, phosphorylated MK2; *blue arrow*, non-phosphorylated MK2. *C*, HEK293 cells were transfected with expression vectors for EGFP-tagged kinase-dead EphA2 (EphA2-KD-EGFP), FLAG-tagged RSK1, FLAG-tagged p38α, MK2 (wild-type (WT) or kinase-dead mutant (KD)), and/or an empty vector. At 24 h post-transfection, whole-cell lysates were immunoblotted with primary antibodies against pS-EphA2, EphA2, pRSK, FLAG, MK2, and β-Actin. *D*, HeLa cells were treated with 10 μM MK2 inhibitor III and then stimulated with 100 μM CDDP for 3 h or 0.3 M NaCl (Osmo) for 10 min. Whole-cell lysates were immunoblotted with primary antibodies against pS-EphA2, EphA2, pRSK, pMK2, and α-Tubulin. *E*, immunoprecipitated RSK1 and RSK2 prepared from HeLa cells were incubated with recombinant human active GST-MK2 at 30 °C for 30 min. Reaction mixtures were analyzed by immunoblotting with anti-phospho-RSK (Ser-380 of RSK1; Ser-386 of RSK2), RSK1, RSK2, and pMK2 antibodies. EphA2, ephrin type-A receptor 2; MK2, MAPK-activated protein kinase 2, RSK, p90 ribosomal S6 kinase.

To investigate the relationship between p38 and RSK, we compared the RSK1 amino acid sequence around Ser-380 and the consensus substrate recognition sequence of p38α (Fig. S2*B*, left). The conserved sequence of p38α substrates is pSer/Thr-Pro (17); however, the corresponding amino acid of RSK1 next to Ser-380 is phenylalanine. Therefore, we focused on MAPK-activated protein kinase 2 (MK2), a main downstream kinase of p38 (18, 19, 20). The sequence around Ser-380 of RSK1 closely matched the substrate recognition sequence of MK2, suggesting that MK2 directly catalyzed RSK phosphorylation (Fig. S2*B*, right). As shown in Figure 3*A*, both EGF and anisomycin induced a shift-up band in the pMK2 blot (red arrow). The shift-up band of pMK2 arises from the use of alternative translation start sites in the mRNA, indicating the activation of MK2 (21). Because the phospho-MK2 antibody used in Figure 3 was unable to distinguish between phospho-MK2 and unphosphorylated MK2 at the endogenous level, activated MK2 is detected as a shift-up band. We also confirmed MK2 activation by detecting the phosphorylation of its established substrate heat shock protein 27 (HSP27) (Fig. S3*A*). MK2 inhibitor or the knockdown of MK2 blocked the anisomycin- but not EGF-induced phosphorylation of RSK and EphA2 (Figs. 3, *A* and *B* and S3*B*). Furthermore, wild-type (WT) MK2, but not kinase-dead MK2, strongly induced the phosphorylation of RSK and EphA2 in HEK293 cells (Fig. 3*C*). Moreover, MK2 inhibitor III blocked the CDDP- or osmotic stress-induced phosphorylation of RSK and EphA2 (Figs. 3*D* and S3*C*). The results of *in vitro* kinase assays showed that recombinant active MK2 catalyzed the phosphorylation of both RSK1 Ser-380 and RSK2 Ser-386 (Fig. 3*E*). These results demonstrated that MK2 activated by p38 directly catalyzed RSK phosphorylation, which resulted in the non-canonical activation of EphA2.

### MK2 induces the atypical activation of RSK

**Figure 4:**
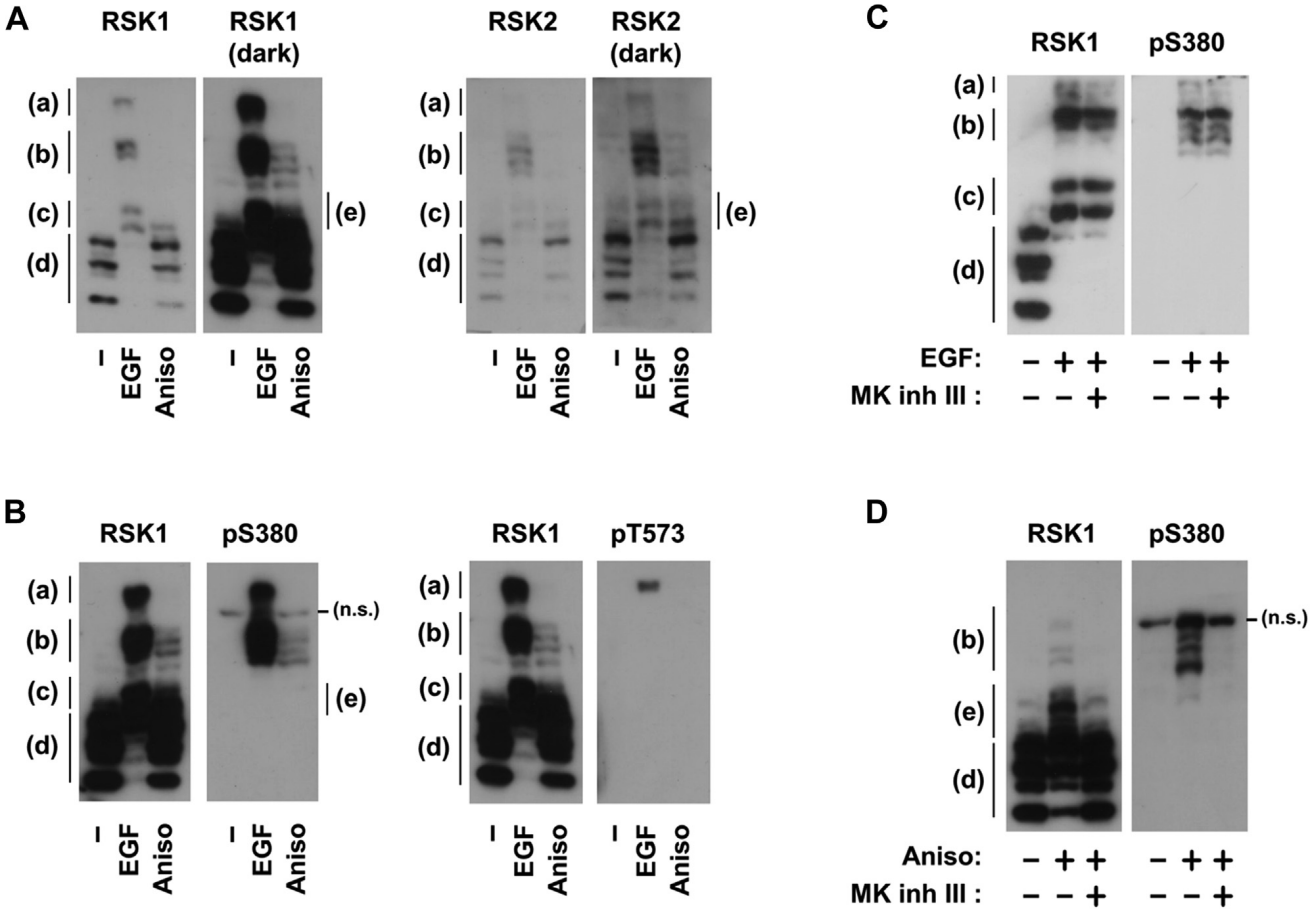
**MK2 induced the atypical activation of RSK.***A* and *B*, HeLa cells were stimulated with 10 ng/ml EGF for 10 min or 50 μM anisomycin for 20 min. *C* and *D*, HeLa cells were treated with 10 μM MK2 inhibitor III for 30 min and then stimulated with 10 ng/ml EGF for 10 min (*C*) or 50 μM anisomycin for 20 min (*D*). Whole-cell lysates were separated by Zn^2+^ Phos-tag SDS-PAGE, followed by immunoblotting with an anti-phospho-RSK (Ser-380 and Thr-573), RSK1, or RSK2 antibody. The images of RSK1 in *A* (*dark*) and *B* (*right*) are from the same blot.

To further investigate the activation mechanisms of RSKs, we employed a Zn^2+^-Phos-tag immunoblot analysis to compare phosphorylation states upon stimulation with EGF or anisomycin. The bandshift of a targeted protein reflects the state of phosphorylation: number and position (22, 23). As shown in Figure 4*A*, basal RSK1 appeared as three main and two additional bands at position (d). Upon the EGF stimulation, all bands completely shifted to positions (a), (b), and (c). In contrast, a modest bandshift was detected with the anisomycin stimulation, in which there were small, but distinct bands at positions (b) and (e). The main bands were still present at the original position (d); however, the lowest band had largely disappeared. These results indicated that the number of phosphorylated RSK proteins induced by anisomycin was less than that by EGF. The bandshift pattern of RSK2 was similar to that of RSK1, indicating the similar regulation of RSK1 and RSK2. In addition, the Ser-380 phosphorylation of RSK1, an indicator of NTK activation, was detected at positions (a) and (b), but not at position (c), (d), or (e) (Fig. 4*B*). On the other hand, phosphorylation at Thr-573, an indicator of CTK activation, was only detected at position (a) (Fig. 4*B*). Most importantly, MK2 inhibitor III blocked the anisomycin-induced bandshift to position (b) containing phosphorylated Ser-380 but did not affect the EGF-induced bandshift (Fig. 4, *C* and *D*). Therefore, EGF induced RSK1 phosphorylation at the residues containing Ser-380 with/without Thr-573 (positions (a) and (b)). In contrast, anisomycin induced phosphorylation at Ser-380 without Thr-573 (position (b)). Collectively, these results suggested that CTK and NTK were both activated by EGF, whereas only NTK was activated by MK2.

**Figure 5:**
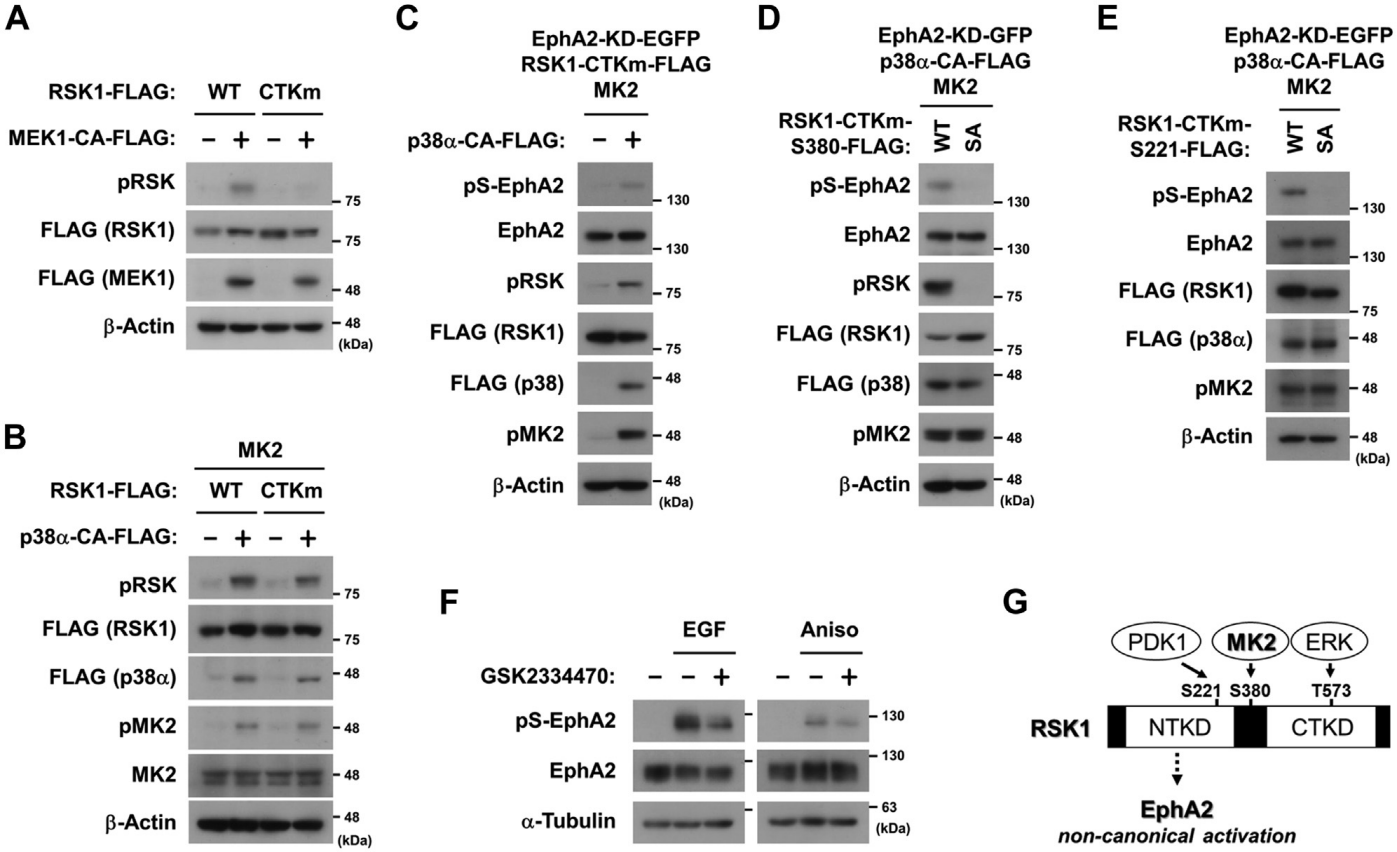
**MK2-induced EphA2 phosphorylation is independent of the CTK activity of RSK.***A* and *B*, HEK293 cells were transfected with the expression vectors for FLAG-tagged RSK1 (wild-type (WT) or CTK-dead mutant (CTKm)), FLAG-tagged constitutively activated MEK1 (MEK1-CA-FLAG), MK2, FLAG-tagged constitutively activated p38α (p38α-CA-FLAG), and/or an empty vector. At 24 h post-transfection, whole-cell lysates were immunoblotted with primary antibodies against phospho-RSK at Ser-380 (pRSK), FLAG, pMK2, MK2, and β-Actin. *C*–*E*, HEK293 cells were transfected with the expression vectors for EphA2-KD-EGFP, RSK1-CTKm-FLAG (Ser-380 WT, Ala-substitute mutation (SA) or Ser-221 SA), MK2, p38α-CA-FLAG, and/or an empty vector. At 24 h post-transfection, whole-cell lysates were immunoblotted with primary antibodies against pS-EphA2, EphA2, pRSK, FLAG, pMK2, and β-Actin. *F*, HeLa cells were treated with 10 μM GSK2334470 for 30 min and then stimulated with 10 ng/ml EGF for 10 min or 50 μM anisomycin for 20 min. Whole-cell lysates were immunoblotted with primary antibodies against pS-EphA2, EphA2 and α-Tubulin. *G*, a schematic diagram of RSK phosphorylation induced by MK2 or ERK. CTK, carboxyl-terminal kinase; EphA2, ephrin type-A receptor 2; MK2, MAPK-activated protein kinase 2; NTK, amino-terminal kinase, RSK, p90 ribosomal S6 kinase.

To confirm that CTK was not necessary, HEK293 cells were transfected with WT RSK1 or a CTK-dead mutant (CTKm). As shown in Figure 5*A*, a constitutively active (CA) mitogen-activated protein kinase 1 (MEK1) (24) induced the Ser-380 phosphorylation of WT RSK1, but not CTKm, indicating the classical ERK-mediated activation of RSK1 in a CTK-dependent manner. In contrast, the constitutively active p38α (CA), in cooperation with MK2-induced RSK1 phosphorylation at Ser-380 of CTKm (Fig. 5*B*), demonstrates that MK2-induced RSK1 phosphorylation at Ser-380 in a CTK-independent manner. This is consistent with the result showing that p38-MK2 signaling promoted the CTKm-mediated phosphorylation of EphA2 (Fig. 5*C*). Furthermore, CTKm with an additional Ser-380 to Ala substitution (SA) failed to promote EphA2 phosphorylation (Fig. 5*D*).

EGF-induced NTK activation is dependent on the phosphorylation of Ser-221 which is catalyzed by PDK1. To determine if Ser-221 phosphorylation is involved in the MK2-promoted activation of RSK-EphA2 axis, HEK293 cells were transfected with the RSK1-CTKm with Ser-221-to-Ala substitution (SA) mutant (Fig. 5*E*). EphA2 was not phosphorylated in the SA mutant expressed cells. In addition, the PDK1 inhibitor GSK2334470 inhibited EphA2 phosphorylation in both EGF- and anisomycin-treated cells (Fig. 5*F*), indicating that Ser-221 phosphorylation by PDK1 is a common event to the NTK activation *via* both the MK2 and ERK signaling pathways.

Taken together, MEK-ERK signaling typically induced the sequential activation of two kinase domains of RSK1: CTK activation by ERK-mediated phosphorylation at Thr-573 followed by NTK activation by CTK-mediated autophosphorylation at Ser-380. On the other hand, MK2 directly phosphorylated RSK1 at Ser-380 without CTK activation to induce atypical NTK activation in the stress-induced non-canonical activation of EphA2. Ser-221 phosphorylation *via* PDK1 is essential for both typical and atypical NTK activation (Fig. 5*G*).

### The p38-MK2-RSK-EphA2 pathway induces cell migration

**Figure 6:**
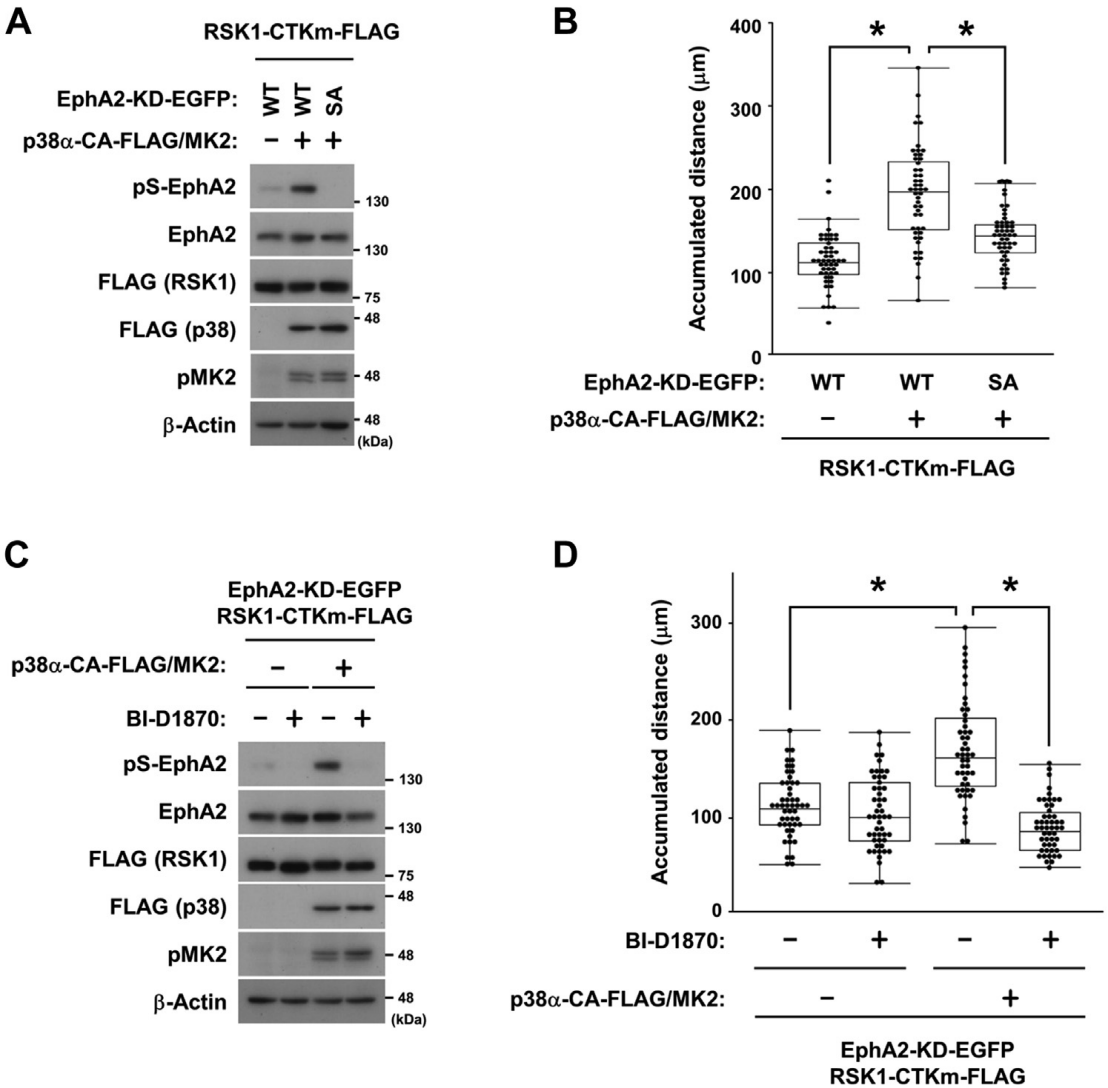
**The p38-MK2-RSK-EphA2 pathway regulates cell migration.** HEK293 cells were transfected with the expression vectors for EphA2-KD-EGFP (Ser-897 WT or SA), RSK1-CTKm-FLAG, p38α-CA-FLAG, MK2, and/or an empty vector. At 24 h post-transfection, cells were treated with DMSO or 10 μM BI-D1870 for 2 h (*C* and *D*). Whole-cell lysates were immunoblotted with primary antibodies against pS-EphA2, EphA2, FLAG, pMK2, and β-Actin (*A* and *C*). Cell migration was observed using a time-lapse imaging system for 120 min (*B* and *D*). The accumulated distance of cell migration (μm) was calculated and shown in *box* and whisker plots. ∗*p* < 0.05 by the Tukey–Kramer HSD test. EphA2, ephrin type-A receptor 2; MK2, MAPK-activated protein kinase 2; RSK, p90 ribosomal S6 kinase.

The RSK-EphA2 pathway is known to promote cell migration (3). Here, we elucidated the contribution of the p38-MK2-RSK-EphA2 pathway in cell migration. RSK1-CTKm and constitutively active (CA) p38 with wild-type (WT) EphA2, but not Ser-897 to Ala substitution (SA), promoted cell migration in HEK293 cells (Fig. 6, *A* and *B*). Moreover, the RSK inhibitor BI-D1870 abolished the non-canonical activation of EphA2 *via* the p38-MK2 axis to suppress cell migration (Fig. 6, *C* and *D*). These results demonstrated the p38-MK2 signaling and the RSK-EphA2 signaling are acting in a linear pathway to promote cell migration.

**Figure 7:**
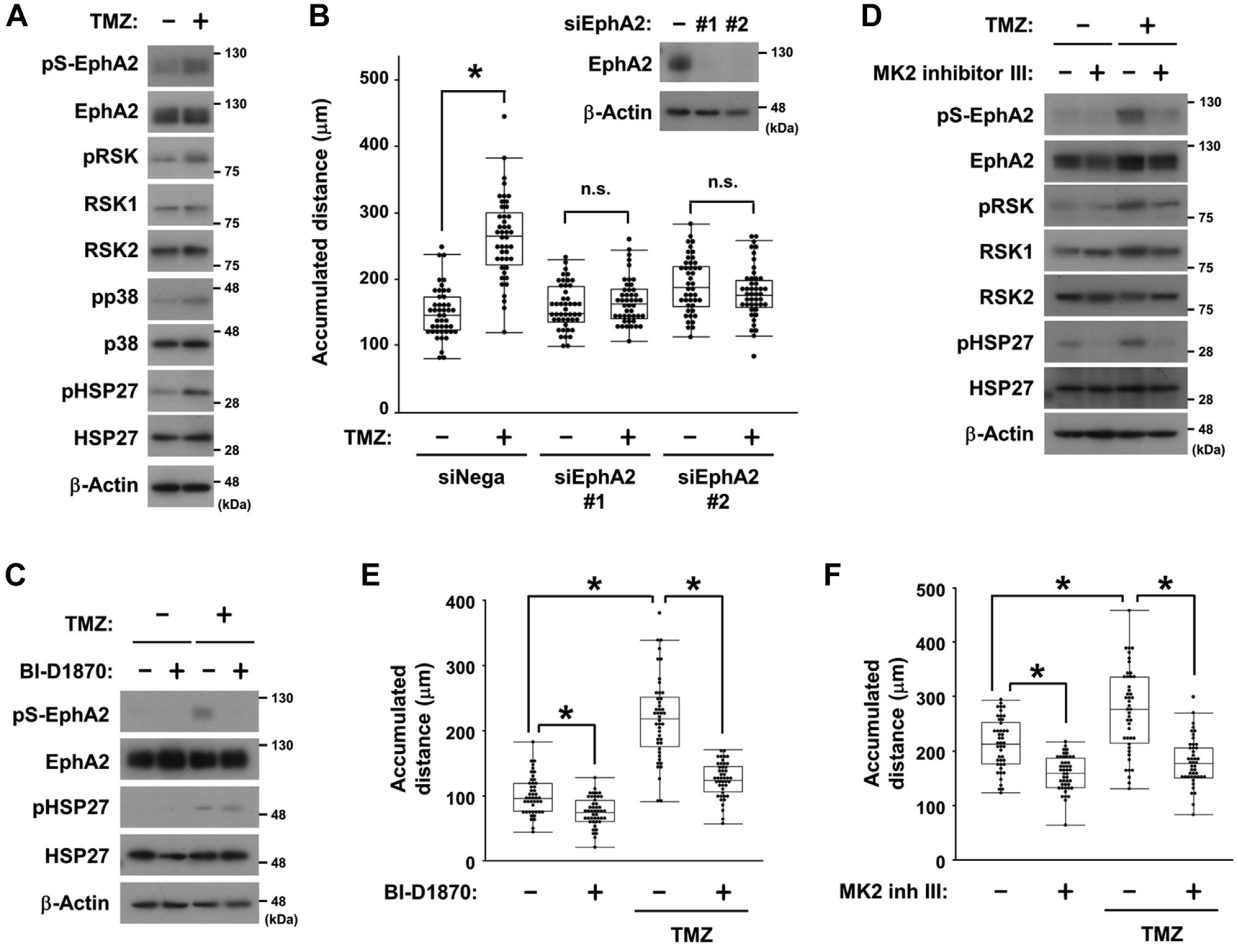
**The p38-MK2-RSK-EphA2 pathway promotes U87-MG cell migration induced by TMZ.***A*, U87-MG cells were treated with DMSO or 100 μM temozolomide (TMZ) for 48 h. Whole-cell lysates were immunoblotted with primary antibodies against pS-EphA2, EphA2, pRSK, RSK1, RSK2, pp38, p38, pHSP27, HSP27 and β-Actin. *B*, U87-MG cells were transfected with siRNA against EphA2 (#1 or #2) or the negative control. After 5 h of transfection, cells were treated with DMSO or 100 μM TMZ for 72 h. Cell migration was observed using a time-lapse imaging system for 120 min. The accumulated distance of cell migration (μm) was calculated and shown in *box* and whisker plots. ∗*p* < 0.05 by the Tukey–Kramer HSD test. Whole-cell lysates were immunoblotted with primary antibodies against EphA2 and β-Actin. *C*–*F*, U87-MG cells were treated with DMSO or 100 μM TMZ for 72 h, then treated with DMSO, 10 μM BI-D1870 (*C* and *E*) or 10 μM MK2 inhibitor III (*D* and *F*) for 2 h. Whole-cell lysates were immunoblotted with primary antibodies against pS-EphA2, EphA2, pRSK, RSK1, RSK2, pHSP27, HSP27, and β-Actin (*C* and *D*). Cell migration was observed using a time-lapse imaging system for 120 min and the accumulated distance of cell migration (μm) was calculated and shown in box and whisker plots (*E* and *F*). ∗*p* < 0.05 by the Tukey–Kramer HSD test. DMSO, dimethyl sulfoxide; EphA2, ephrin type-A receptor 2; HSP27, heat shock protein 27; RSK, p90 ribosomal S6 kinase.

Next, we used human glioblastoma U87-MG cells because treatment with the alkylating chemotherapeutic agent temozolomide (TMZ) provokes cellular stress to induce p38 activation as well as the migratory ability (25). TMZ activated MK2 by detecting phosphorylation of HSP27 to enhance the RSK-EphA2 axis in U87-MG cells (Figs. 7*A* and S4*A*). TMZ-induced cell migration was inhibited by the knockdown of EphA2 using small interfering RNA (siRNA) (Fig. 7*B*). RSK inhibitors blocked EphA2 phosphorylation but not HSP27 phosphorylation, whereas MK2 inhibitors blocked both EphA2 and HSP27 phosphorylation (Figs. 7, *C* and *D* and S4*B*). In addition, both RSK and MK2 inhibitors suppressed cell migration (Figs. 7, *E* and *F* and S4, *C*–*E*). These results demonstrated that the p38-MK2-RSK-EphA2 pathway regulated cell migration under stress conditions.

## Discussion

**Figure 8:**
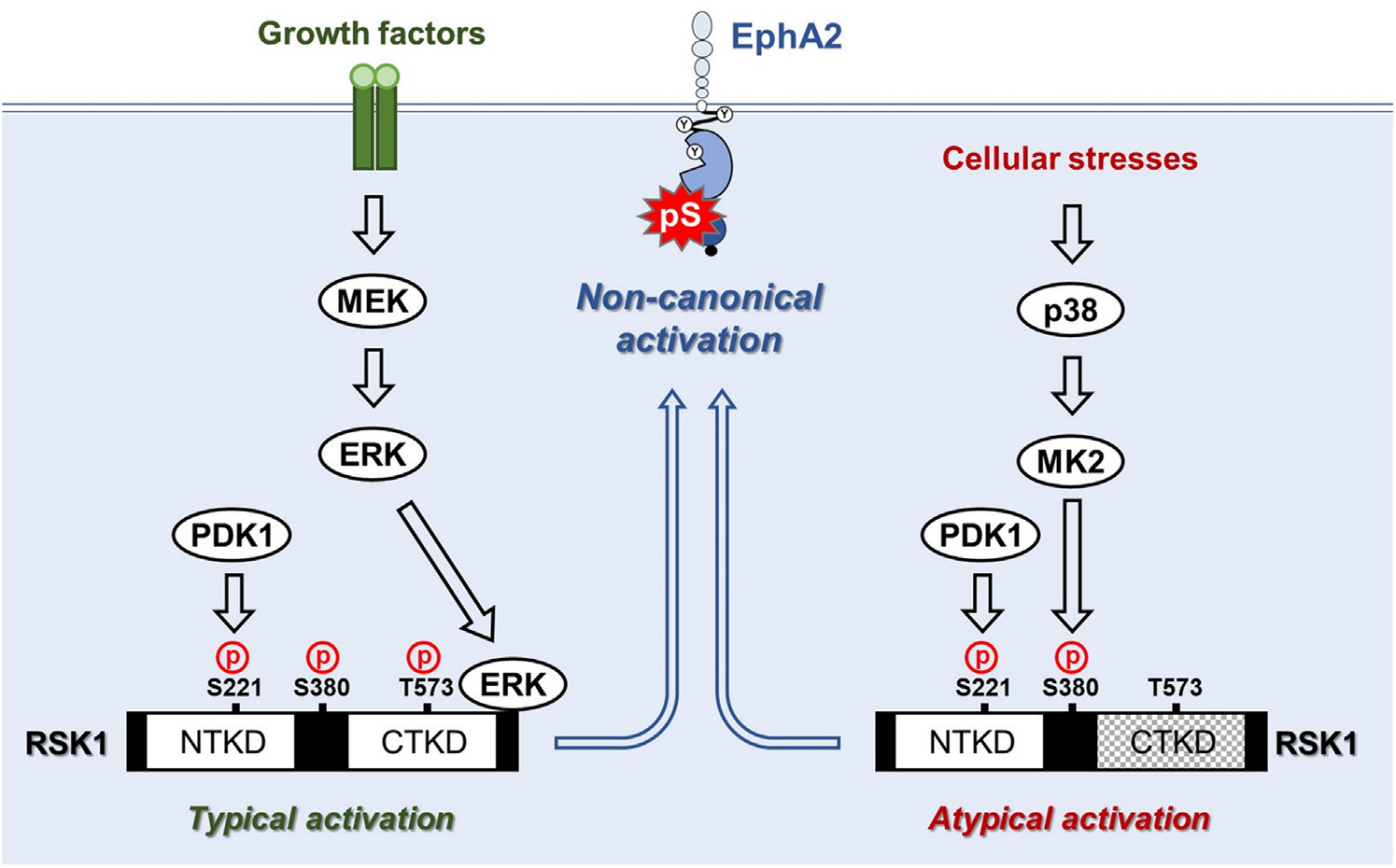
**Model of the non-canonical activation of EphA2 by typically or atypically activated RSK.** Upon the stimulation of growth factors, ERK typically activates CTK to induce the activation of NTK, resulting in the non-canonical activation of EphA2. On the other hand, cellular stress-activated MK2 catalyzes the phosphorylation of RSK1 at Ser-380 to induce NTK activation in a CTK-independent manner. This atypically activated RSK also induces the non-canonical activation of EphA2 to promote cancer malignancy. CTK, carboxyl-terminal kinase; EphA2, ephrin type-A receptor 2; ERK, extracellular signal-regulated kinase; MK2, MAPK-activated protein kinase 2; NTK, amino-terminal kinase; RSK, p90 ribosomal S6 kinase.

In the present study, we identified a novel molecular mechanism of non-canonical EphA2 activation (Fig. 8). We and others reported that non-canonical EphA2 activation is induced by the MEK-ERK-RSK pathway (3). In the present study, we demonstrated for the first time that the p38-MK2-RSK pathway mediated the stress-induced non-canonical activation of EphA2, in which MK2 catalyzed the phosphorylation of RSK1/2 at Ser-380/386 to induce PDK1- and phosphorylation of Ser-221/227-dependent NTK activation in a CTK-independent manner. This novel p38-MK2-RSK-EphA2 pathway also controlled cancer cell migration under stress conditions in TMZ-treated human glioblastoma U87-MG cells.

The constitutive non-canonical activation of EphA2 *via* the MEK-ERK pathway regulates the malignant progression of various tumors, including cancer cell motility, EMT, stemness properties, and drug resistance (3). Cancer cells in the tumor microenvironment are exposed to cellular stresses, including oxidative stress, chronic inflammation, and therapeutic anti-cancer agents (26, 27). Under these conditions, p38 induces EMT to promote tumor metastasis and control the self-renewal of lung stem cells by inhibiting proliferation and differentiation (14, 15). Since p38 is strongly activated in chemo-resistant cancer cells, a p38 inhibitor may overcome this resistance. The most important point of the present results is that these pro-tumor functions may be regulated by stress-induced non-canonical EphA2 activation *via* the p38-MK2 pathway, possibly in cooperation with ERK-mediated EphA2 activation.

Under stress conditions, p38 is known to activate not only MK2 but also mitogen- and stress-activated kinase (MSK). MSK belongs to the AGC subfamily of protein kinases along with RSK. Interestingly, other AGC subfamily protein kinases AKT and PKC have also been reported to catalyze EphA2 phosphorylation at Ser-897 (8, 9). Thus, we tried to elucidate the contribution of MSK in the p38-RSK-EphA2 axis. Although MSK and RSK belong to the same subfamily, their consensus sequences for substrate recognitions are different (Fig. S5*A*). The conserved sequence of MSK1 substrates is Arg-X-pSer; however, the corresponding amino acid of EphA2 near Ser-897 is Leu-Pro-pSer, suggesting that MSK does not catalyze EphA2 phosphorylation. Of note, it also does not match the RSK1 amino acid sequence around Ser-380 (Fig. S5*B*). Taken together, MSK is not involved in the p38-activated MK2-RSK-EphA2 signaling.

MK2 controls various cellular events, including inflammatory responses, cell motility, the cell cycle, and apoptosis, by regulating mRNA stability and protein expression (28, 29). Some reports clarified the biological significance of MK2 in cancer progression including in breast cancer, colorectal cancer, and head and neck squamous cell carcinoma; however, the contribution of MK2 in cancer motility remains largely unknown (30, 31, 32, 33, 34). Previous studies indicated that HSP27 played a crucial role in cell motility (19, 35, 36). HSP27 has also been shown to function as an F-actin capping protein that inhibits actin polymerization (37). Therefore, its phosphorylation by MK2 blocks its capping activity, leading to the promotion of cell migration with the remodeling of the actin cytoskeleton. Furthermore, the p38-MK2-HSP27 axis was found to promote the invasion of prostate and bladder cancer cells, in which the stress signaling pathway regulates mRNA stability and the activities of the matrix metalloproteinases (MMP)-2 and MMP-9 (38). Hence, under cellular stress conditions, MK2 regulates non-canonical EphA2 as well as HSP27, which may synergistically control cellular motility. HSP27 inhibitor J2 did not inhibit TMZ-induced U87-MG cell migration (Fig. S6), indicating that the non-canonical activation of RSK-EphA2 axis, but not HSP27, regulates TMZ-induced cell migration.

MK2 has been reported to control the cell cycle by regulating CDC25B and CDC25C phosphatases (20, 39, 40). CDC25B/C function as G2/M checkpoint regulators that induce the activation of cyclin-dependent kinase 1 (CDK1) to promote the transition from the G2 phase to the M phase. In response to DNA damage, CDC25B/C are phosphorylated by checkpoint kinase 2 and transferred from the nucleus to the cytoplasm, where they fail to activate CDK1. Manke *et al.* (20) demonstrated that UV-induced DNA damage resulted in G2/M arrest by promoting the phosphorylation of CDC25B and CDC25C by MK2. They also found that MK2 was necessary for S phase arrest and the knockdown of MK2-sensitized cells to DNA damage-induced cell death. Therefore, MK2 may be a member of the DNA damage response kinase family. On the other hand, EphA2 is known to be involved in cell cycle regulation in normal cells, with the activation of the non-canonical RSK-EphA2 pathway by CDK1 controlling M-phase progression, particularly mitotic entry (41). pS-EphA2 was shown to promote mitotic spindle formation by maintaining cortical rigidity, indicating its crucial involvement in the cell cycle. In contrast, the function of pS-EphA2 in the cell cycle following DNA damage remains unknown; therefore, further studies are needed to clarify the functions of the p38-MK2-RSK-EphA2 axis in the cell cycle.

Cellular stress promotes the activation of the p38-MK2 axis to induce the atypical activation of RSK (42, 43). RSK has many substrates that regulate various physiological phenomena, including cell growth, anti-apoptosis, and protein synthesis (12, 13). Zaru *et al.* reported that MK2 induced the atypical activation of RSK in dendritic cells and macrophages stimulated by toll-like receptor ligands (42, 43). They also examined the effects of EGF, anisomycin, and 12-*O*-tetradecanoylphorbol 13-acetate (TPA) in B cells, T cells, and fibroblasts; however, MK2 was dispensable for the activation of RSK. In the present study, we found that anisomycin, CDDP, and high osmotic stress selectively promoted p38-MK2-mediated atypical RSK activation in HeLa cells (Fig. 3*D*). In contrast, similar to the findings reported by Zaru *et al.*, only the typical activation of RSK was induced by EGF and TPA, possibly due to the earlier and stronger activation of ERK than anisomycin (Figs. 3*B* and S7). Therefore, the intensity and onset of activation of ERK and MK2, two independent upstream activators of RSKs, affects the balance between typical and atypical activation. CDDP strongly activated the p38-MK2 pathway at 3 h, and weakly activated the ERK pathway at 6 h, which correlated with atypical RSK activation at 3 h and subsequent canonical activation at 6 h (data not shown). These results suggest that while ERK preferentially activates RSK, atypical activation is temporarily driven to rapidly trigger their cellular stress responses *via* the p38-MK2 pathway, for example, to escape from the cytotoxicity of anti-cancer agents.

We previously demonstrated that treatment with trametinib, a MEK inhibitor, effectively down-regulated the RSK-EphA2 pathway in cancer cell lines with constitutive ERK activation (7). The p38-MK2 axis is activated by cellular stresses in the tumor microenvironment *in vivo*, which may cause the atypical activation of the RSK-EphA2 pathway. Therefore, combination therapy of trametinib with a p38-MK2 inhibitor has the potential to completely block the non-canonical activation of EphA2. Preclinical studies utilizing p38 inhibitors have been successful (14); however, none have been clinically approved due to their adverse effects. The MK2 inhibitor ATI-450 (also known as CDD-450) was safe and well tolerated in a Phase 1 clinical trial, and, thus, Phase 2a trials are forthcoming (44). Current therapeutic indications for ATI-450 are rheumatoid arthritis, hidradenitis suppurativa, and psoriatic arthritis. The present results on atypical EphA2 activation suggest the potential of ATI-450 in the treatment of various tumors in combination with trametinib.

In conclusion, we herein demonstrated that the p38-MK2 axis induced the atypical activation of RSK to promote the non-canonical activation of EphA2 under cellular stress conditions. The present results clarified the relationship between the p38-MK2 axis and EphA2 non-canonical activation and provide an effective strategy for anti-EphA2 therapy to prevent the malignant progression of human cancers.

## Experimental procedures

### Antibodies and reagents

Total antibodies against EphA2 (#6997) and phospho-specific antibodies against EphA2 (Ser-897; #6347), RSK (Ser-380; cross-reacting with RSK2 Ser-386; #11989), RSK (Thr-573; #9346), p38 (Thr-180/Tyr-182; #4511), ERK (Thr-202/Tyr-204; #9101), and MK2 (Thr-334; #3041 for Figs. 3 and 5, *C*–*E* or #3007 for Figs 5*B* and 6) were purchased from Cell Signaling Technology; antibodies against total ERK (C-9), p38α (A-12), RSK1 (C-21), RSK2 (C-19), MK2 (A-11), HSP27 (F-4), α-Tubulin (B-7), and β-Actin (C-4) were from Santa Cruz Biotechnology; an antibody against FLAG (F1804) was from Merck KGaA; a phospho-antibody against HSP27 (MAB23141) was from R&D Systems. Recombinant human EGF was obtained from R&D Systems; recombinant human active GST-MK2 protein was from Carna Biosciences; a phos-tag ligand, anisomycin, NaCl, and CDDP were from Wako Pure Chemical Industries; SB203580 and TMZ were from Merck KGaA; MK2 inhibitor III and GSK2334470 were from MedChemExpress; BI-D1870 was from BioVision; BIRB796, Skepinone-L, and BX-702 were from Selleck Chemicals. All chemical inhibitors were dissolved in dimethyl sulfoxide (DMSO).

### Cell cultures

HeLa and HEK293 cells (ATCC) were cultured in Dulbecco’s Modified Eagle’s medium (DMEM) supplemented with 10% fetal calf serum (Merck KGaA), 2 mM L-glutamine (Thermo Fisher Scientific), 100 U/ml penicillin (Meiji Seika Pharma Co, Ltd), and 100 μg/ml streptomycin (Meiji Seika Pharma Co, Ltd) at 37 °C in 5% CO_2_. U87-MG cells (provided by Dr Tsuneo Imanaka (University of Toyama)) were cultured in Eagle’s Minimum Essential medium (Nissui) supplemented with 10% fetal calf serum, 2 mM L-glutamine, 100 U/ml penicillin, and 100 μg/ml streptomycin at 37 °C in 5% CO_2_. Before drug treatment, the culture medium of HeLa cells was changed to DMEM with 0.5% fetal calf serum, 2 mM L-glutamine, 100 U/ml penicillin, and 100 μg/ml streptomycin, and then cells were incubated at 37 °C in 5% CO_2_ for 16 to 24 h.

### Immunoblotting

Whole-cell lysates, prepared as previously described (7), were resolved using SDS-PAGE and transferred to an Immobilon-P nylon membrane (Merck KGaA). The membrane was treated with BlockAce (KAC Co, Ltd) and probed with the primary antibody at room temperature. Antibodies were detected using horseradish peroxidase-conjugated anti-rabbit, anti-goat, or anti-mouse IgG (DAKO) diluted in Can Get Signal solution (TOYOBO) or PBS containing 0.1% Tween 20 (Wako Pure Chemical Industries). Signals were detected with an enhanced chemiluminescence system (Thermo Fisher Scientific). Analysis was carried out at least three times, and representative results are shown.

### Zn^2+^ phos-tag SDS-PAGE

Whole-cell lysates were harvested with RIPA buffer and Zn^2+^ Phos-tag SDS-PAGE was performed according to a previously reported procedure (7). After SDS-PAGE, the gel was washed twice using a solution containing 25 mM Tris, 192 mM glycine, 10% methanol, and 1.0 mM EDTA and once using a solution containing 25 mM Tris, 192 mM glycine, and 10% methanol. Gel transfer, membrane blocking, antibody reactions, and signal detection were conducted according to the normal immunoblotting procedure. Analysis was carried out at least three times and representative results are shown.

### RNA interference

siRNAs were synthesized at Thermo Fisher Scientific (Stealth RNA interference) or Hokkaido System Science. Target sequences were as follows: 5′-GCAUUACAACCAGACAGUUGAUAUU-3′ (p38α), 5′-CCAGUAUGAAUUUCCCAACCCAGAA-3′ (MK2), 5′-UGGAGUCCAUCAAGAUGCAGCAGUA-3′ (EphA2 #1), 5′-GCAAGGAAGUGGUACUGCUGGACUU-3′ (EphA2 #2) and 5′-UAAUGUACUGCGCGUGGAGAGGAA-3′ (negative control). HeLa cells were transfected with siRNAs at a final concentration of 20 to 100 nM using Lipofectamine RNAiMAX (Thermo Fisher Scientific) in accordance with the manufacturer’s instructions.

### Transfection of plasmid DNAs

The expression vector for the human kinase-dead (KD) mutant EphA2 was provided by Dr Haruhiko Sugimura (Hamamatsu University School of Medicine, Hamamatsu, Japan) (45, 46). The EGFP-tagged EphA2-KD expression plasmid was generated using NEBuilder HiFi DNA Assembly Master Mix (New England BioLabs). Human p38α and MK2 (the canonical short isoform) cDNA were amplified from HeLa cells by RT-PCR using KOD DNA Polymerase and KOD -Plus- Neo polymerase (Toyobo), respectively. Human p38α cDNA was subcloned into the BglII-SmaI site in pFLAG-CMV-2 vector using Ligation high (Toyobo). Human MK2 cDNA was inserted into the pcDNA3.1 vector using NEBuilder HiFi DNA Assembly Master Mix. The expression vectors for FLAG-tagged human RSK1 and MEK1 were provided by Dr Yoshikazu Sugimoto (Keio University, Tokyo, Japan) (47). The expression vector for kinase-dead (KD) mutation (K53M) of p38α was introduced using the QuikChange kit (Agilent Technologies, Inc) in accordance with the manufacturer’s instructions. The expression vectors for CTK-dead (CTKm: K447R), CTKm-S221A, and CTKm-S380A mutants of RSK1, constitutively active (CA) mutations of p38α (D176A and F327L), and MEK1 (Q56P), kinase-dead (KD) mutant of MK2 (K94M) were generated by RT-PCR with KOD -Plus- Neo polymerase. HEK293 cells were transfected using Lipofectamine 2000 (Thermo Fisher Scientific) in accordance with the manufacturer’s instructions.

### *In vitro* kinase assay

The RSK1 and RSK2 proteins were obtained by immunoprecipitation. Whole-cell lysates were described earlier. Lysates were incubated with anti-RSK1 or RSK2 antibodies at 4 °C overnight and then rotated with Dynabeads protein G (Thermo Fisher Scientific) at 4 °C for 1.5 h. Beads were washed three times with PBS containing 0.1% Tween 20. Recombinant human GST-MK2 (50 ng) was then added and reacted at 30 °C for 30 min in 30 ml of reaction buffer containing 20 mM HEPES (pH 7.6), 20 mM MgCl_2_, 0.2 mM ATP, 2 mM DTT, 20 mM β-glycerophosphate, and 0.1 mM sodium orthovanadate. After stopping the reaction with the addition of 30 μl of SDS–PAGE sample buffer, immunoblotting was performed as described above.

### Migration assay

Assay was performed as described previously (25). Briefly, cells were seeded on a 24-well plate. After drug treatment, cell migration was observed using a time-lapse live cell imaging system (Cell Observer, Carl Zeiss). Images were captured every 10 min for 120 min. The accumulated migrating distance of 45 to 50 cells was calculated by ImageJ/Fiji software (National Institute of Health) and analyzed by Chemotaxis and Migration Tool (ibidi GmbH). Statistical analyses were performed using JMP software version 11 (SAS Institute Japan). Assay values are given as the mean ± SD. The significance of differences was analyzed by the Tukey–Kramer HSD test. Probability values of *p* < 0.05 were considered to be significant. Similar results were obtained in at least three independent experiments.

## Data availability

All data are contained in this article.

## Supporting information

This article contains supporting information (17).

## Conflicts of interest

The authors declare that they have no conflicts of interest with the contents of this article.
