# Supplementary material for: Cellular stress induces non-canonical activation of the receptor tyrosine kinase EphA2 through the p38-MK2-RSK signaling pathway
Source: J Biol Chem. 2023 Apr 12;299(5):104699. doi: 10.1016/j.jbc.2023.104699 (PMC10196987; doi:10.1016/j.jbc.2023.104699)
Supplement: Supporting Figures S1–S7 [file mmc1.pdf]

Supplementary Figure S1

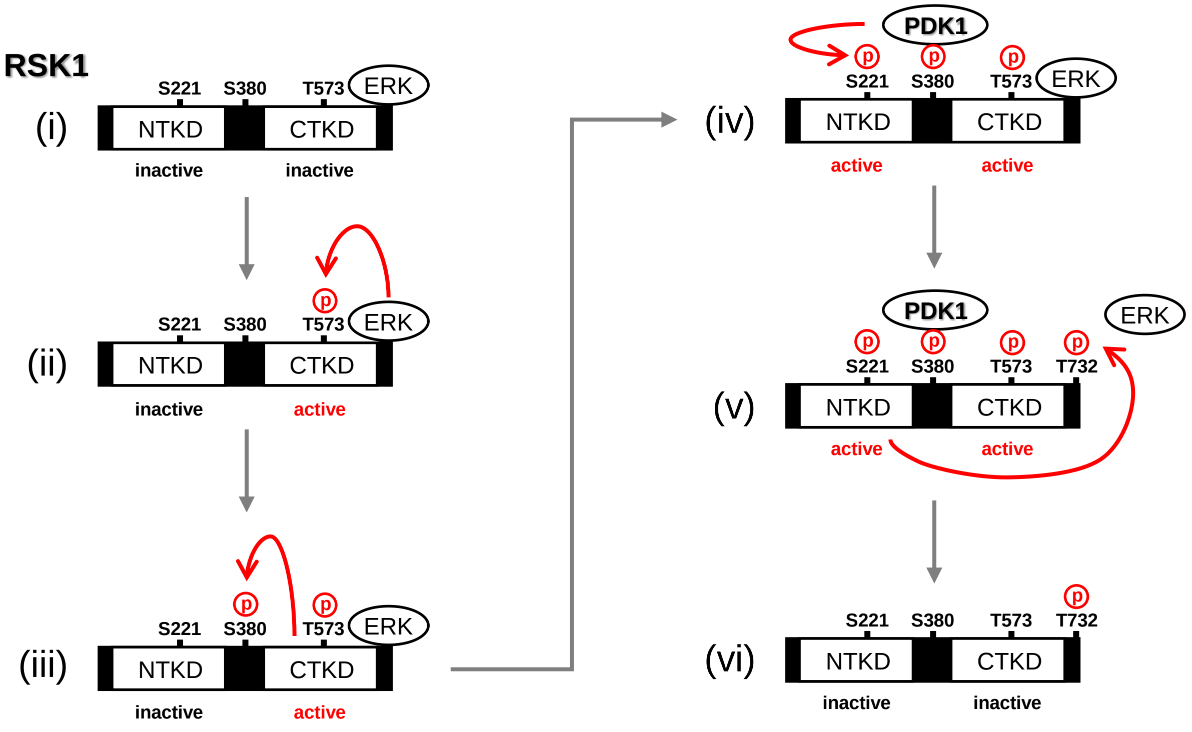

Supplementary Figure S1. The activation and inactivation mechanisms of RSK1.

(i) Active ERK binds to the docking site in the carboxyl-terminal.

(ii) ERK phosphorylates Thr-573 to induce the activation of CTK.

(iii) RSK1-CTK auto-phosphorylates Ser-380.

(iv) PDK1 binds to phosphorylated Ser-380 and catalyzes the phosphorylation at Ser-221 to promote the activation of NTK.

(v) NTK induces the phosphorylation at Thr-732, resulting in the detachment of ERK.

(vi) Phosphatases dephosphorylate the phosphorylation to inactivate RSK1.

Supplementary Figure S2

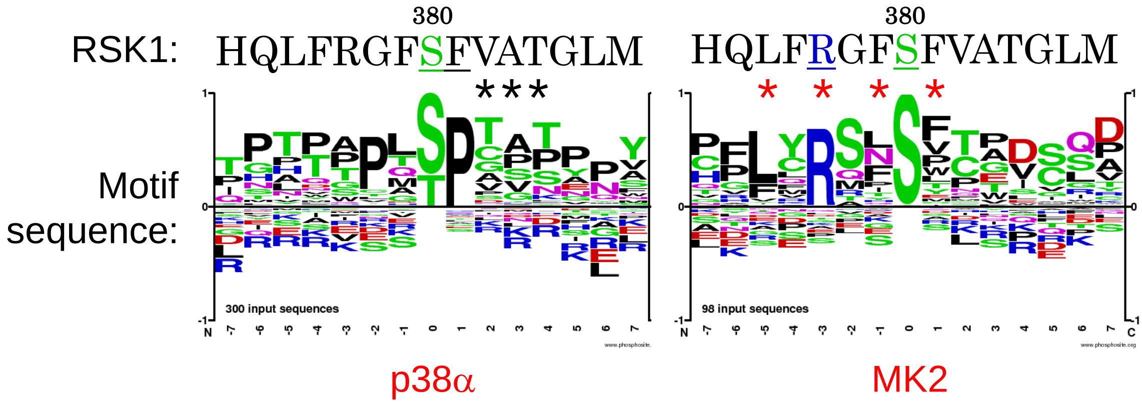

Supplementary Figure S2. MK2 controls RSK phosphorylation to regulate the phosphorylation of EphA2 at Ser-897.

Comparison of RSK1 amino acid sequence around Ser-380 with the substrate sequence motifs of p38α and MK2. The substrate sequence motifs were obtained from PhosphoSitePlus (17).

Supplementary Figure S3

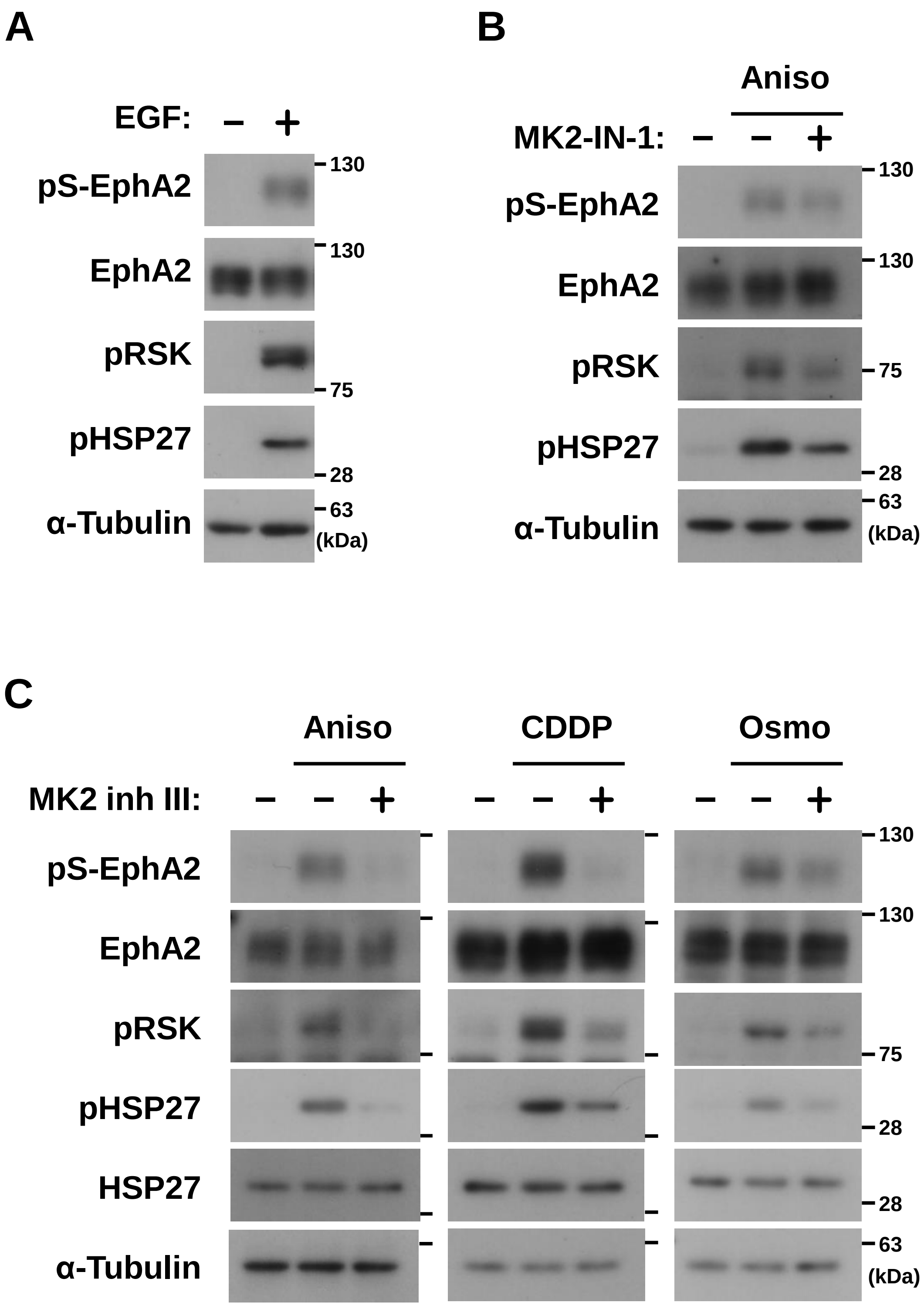

Supplementary Figure S3. MK2 controls RSK phosphorylation to regulate the phosphorylation of EphA2 at Ser-897.

A, HeLa cells were treated with 100 ng/mL EGF for 10 min. *B*, HeLa cells were treated with 10 μM MK2-IN-1 for 30 min and then stimulated with 50 μM anisomycin for 20 min. *C*, HeLa cells were treated with 10 μM MK2 inhibitor III and then stimulated with 50 μM anisomycin for 20 min, 100 μM CDDP for 3 h or 0.3 M NaCl for 10 min. Whole-cell lysates were immunoblotted with primary antibodies against pS-EphA2, EphA2, pRSK, pHSP27, HSP27, and α-Tubulin.

Supplementary Figure S4

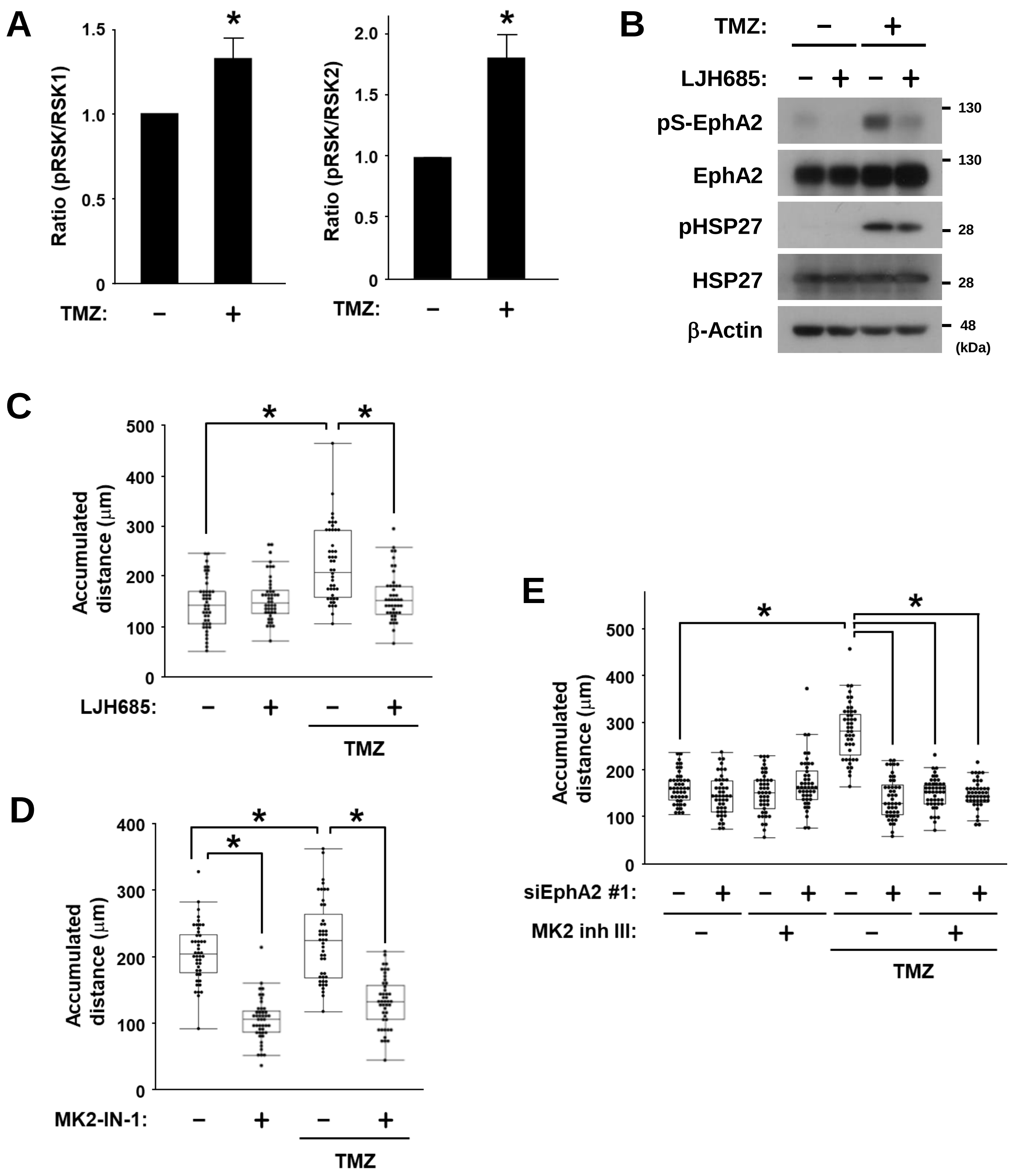

Supplementary Figure S4. TMZ induces RSK phosphorylation and cell migration via the MK2-RSK pathway.

A, Quantitative analysis of the immunoblotting data. The ratio of pRSK/RSK1 and pRSK/RSK2 of three replicates was presented. Values are given as the mean ± SD. **P*<0.05 by Student's *t*-test. *B*, U87-MG cells were treated with DMSO or 100 μM temozolomide (TMZ) for 72 h, then treated with DMSO or 50 μM LJH685 for 2 h. Whole-cell lysates were immunoblotted with primary antibodies against pS-EphA2, EphA2, pHSP27, HSP27 and α-Tubulin. *C* and *D*, U87-MG cells were treated with DMSO or 100 μM temozolomide (TMZ) for 72 h, then treated with DMSO, 50 μM LJH685 (*C*) or 10 μM MK2-IN-1 (*D*) for 2 h. *E*, 87-MG cells were transfected with siRNA against EphA2 (#1) or the negative control. After 5 h of transfection, cells were treated with DMSO or 100 μM temozolomide (TMZ) for 72 h, then treated with DMSO, 10 μM MK2 inhibitor III for 2 h. Cell migration was observed using a time-lapse imaging system for 120 min. The accumulated distance of cell migration (μm) was calculated and shown in box and whisker plots. **P*<0.05 by the Tukey-Kramer HSD test.

Supplementary Figure S5

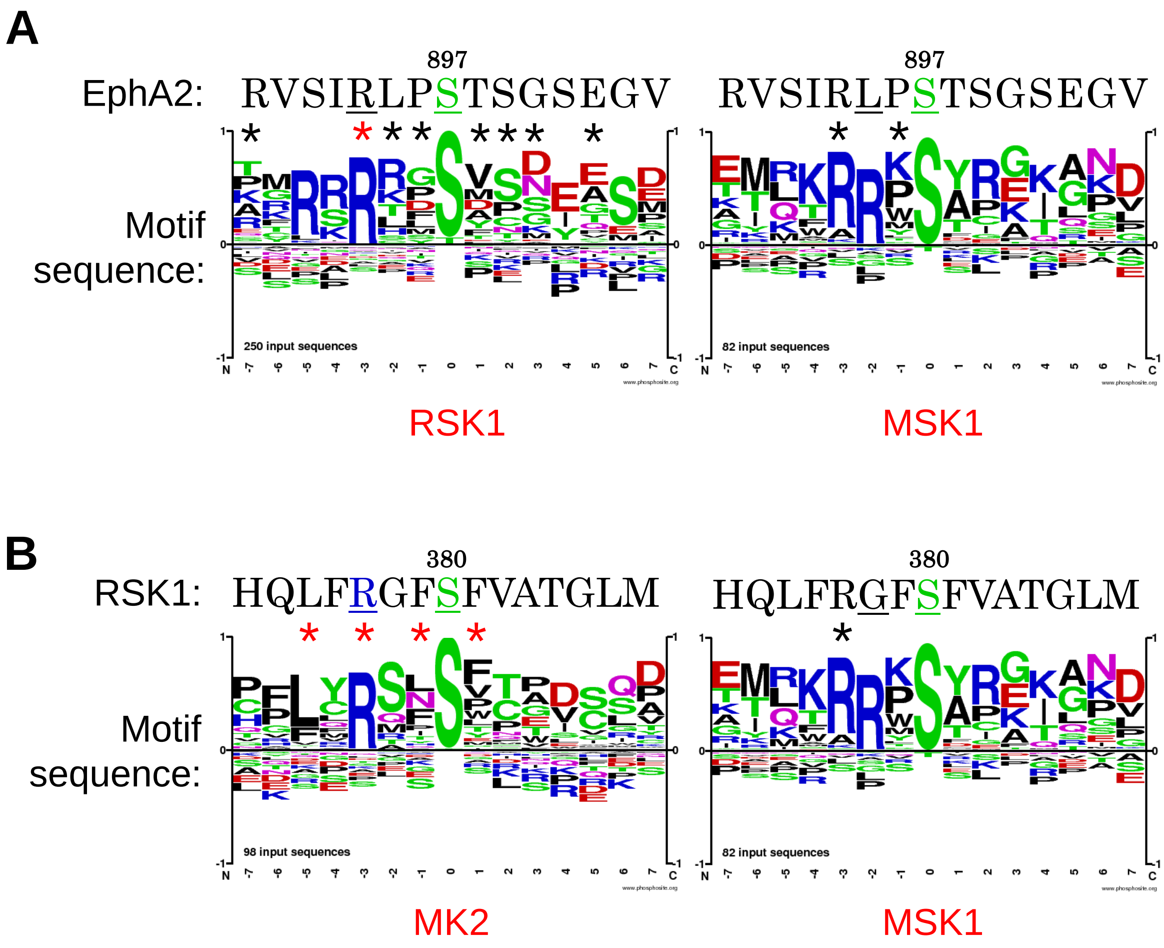

Supplementary Figure S5. MSK has no contribution to the RSK-EphA2 pathway.
A, Comparison of EphA2 amino acid sequence around Ser-897 with the substrate sequence motifs of RSK1 and MSK1. *B*, Comparison of RSK1 amino acid sequence around Ser-380 with the substrate sequence motifs of MK2 and MSK1. The substrate sequence motifs were obtained from PhosphoSitePlus (17).

Supplementary Figure S6

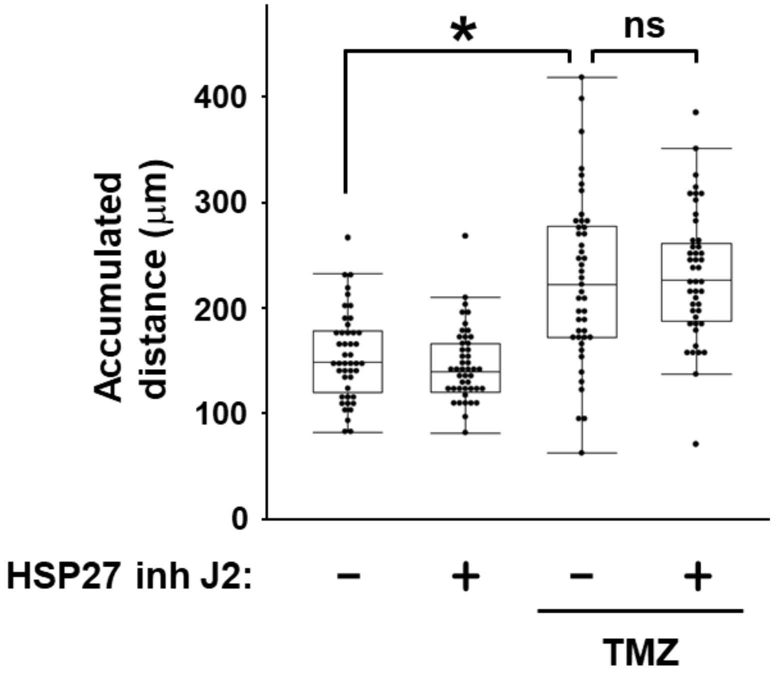

Supplementary Figure S6. TMZ induces cell migration independent of HSP27.
U87-MG cells were treated with DMSO or 100 μM temozolomide (TMZ) for 72 h, then treated with DMSO or 10 μM HSP27 inhibitor J2 for 2 h. Cell migration was observed using a time-lapse imaging system for 120 min. The accumulated distance of cell migration (μm) was calculated and shown in box and whisker plots. *$P<0.05$ by the Tukey-Kramer HSD test.

Supplementary Figure S7

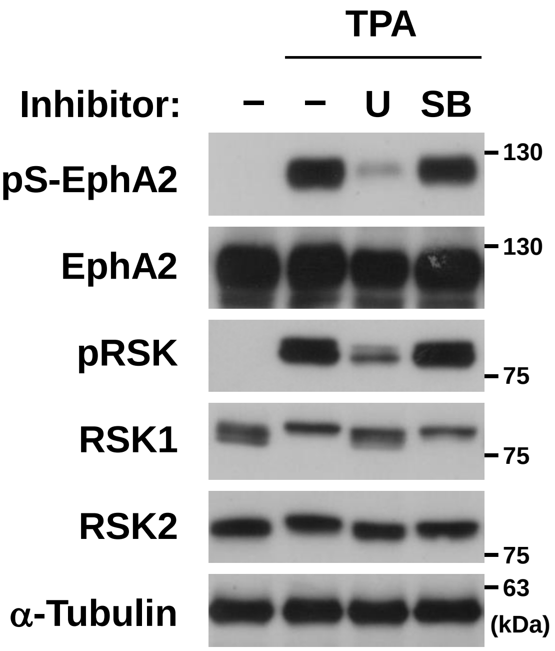

Supplementary Figure S7. TPA induces the RSK-EphA2 pathway not via p38.
HeLa cells were treated with 10 μM U0126 (U) or SB203580 (SB) for 30 min, and then stimulated with 100 nM TPA for 10 min. Whole-cell lysates were immunoblotted with primary antibodies against pS-EphA2, EphA2, pRSK, RSK1, RSK2, and α-Tubulin.

Supplementary EXPERIMENTAL PROCEDURES

Reagents

RSK inhibitor LHJ685, MK2 inhibitor MK2-IN-1, and HSP27 inhibitor J2 were obtained from MedChemExpress; 12-O-tetradecanoylphorbol 13-acetate (TPA) was from Wako Pure Chemical Industries.
